# Seizures Following Single-Dose Intravenous Tramadol for Postsurgical Pain: A Report of Two Cases

**DOI:** 10.7759/cureus.93354

**Published:** 2025-09-27

**Authors:** Arya Babul, Aidan Gill, Sohi Ashraf, Momina Hussain, Najib Babul

**Affiliations:** 1 Biomedical Sciences, West Career and Technical Academy, Las Vegas, USA; 2 Biomedical Sciences, Society for Awareness of Neglected Diseases, Las Vegas, USA; 3 Medical Affairs, APG Pharma Consulting GmbH, Zug, CHE; 4 Intensive Care, Mountain View Hospital, Las Vegas, USA; 5 Genomics, Chinese Academy of Tropical Agricultural Sciences, Sanya, CHN; 6 Drug Development, Cinergen, LLC, Nevada, USA

**Keywords:** acute pain, analgesia, ketoprofen, molar extraction, morphine, postoperative pain, randomized controlled trial, seizures, tramadol

## Abstract

Two subjects receiving a single intravenous (IV) dose of tramadol 150 mg for severe postsurgical pain developed seizures within four minutes of drug administration despite rigorous screening and exclusion of patients at risk for seizures. Both subjects were participating in a randomized, double-blind study comparing the analgesic efficacy, dose-response relationship, duration of action, and safety of IV ketoprofen 50 mg and 100 mg, IV tramadol 100 mg and 150 mg, IV morphine 4 mg, and matching placebo administered over two minutes for moderate or severe postsurgical pain after removal of two or more third molars, including at least one mandibular, bony impacted third molar. The seizures were deemed to be life-threatening by the investigator and led to termination of the IV tramadol 150 mg treatment arm. Importantly, for all analgesic efficacy outcomes, IV tramadol 150 mg was neither statistically nor numerically superior to IV tramadol 100 mg; however, it was associated with more frequent and severe adverse events. IV ketoprofen 50 mg and 100 mg consistently outperformed IV tramadol 100 mg and 150 mg; the efficacy of IV tramadol at both doses was approximately half that provided by IV ketoprofen; adverse events after single doses of ketoprofen were mild or moderate and comparable to placebo. The occurrence of seizures in two subjects (9% of participants receiving IV tramadol at 150 mg) shortly after drug administration, despite rigorous screening for seizure risk, suggests that for postsurgical pain, even a single bolus dose of IV tramadol 100 mg may provide an insufficient safety margin in the real-world clinical setting. Accordingly, we recommend that IV tramadol for postsurgical pain be administered to adults either as a low-dose, repeated bolus (e.g., 25 to 50 mg every five to 15 minutes) or via a controlled infusion when doses exceed 50 mg, preferably in combination with a mechanistically differentiated analgesic.

## Introduction

Intravenous (IV) tramadol is used outside North America for postsurgical pain, either as monotherapy or in combination with nonsteroidal anti-inflammatory drugs (NSAIDs) to provide multimodal analgesia [1-3]. Its widespread availability as an injectable in more than 135 countries reflects its dual mechanism as a weak µ-opioid agonist and a modest norepinephrine and serotonin reuptake inhibitor. In addition, upon commercialization and for several decades thereafter, tramadol was not subject to controlled substance regulations [4-5]. 

We would like to report two cases of seizures after a single IV dose of tramadol for the management of postsurgical pain. The consenting subjects were participating in a randomized, double-blind, placebo-controlled study comparing the analgesic efficacy, dose-response relationship, duration of action, and safety of IV ketoprofen 50 mg and 100 mg, IV tramadol 100 mg and 150 mg, IV morphine 4 mg, and placebo administered over two minutes for moderate or severe postsurgical pain after removal of ≥2 third molars, including at least one mandibular, bony impacted third molar.

The study was conducted at the Eastman Dental Institute Research Center (EDI), University College Hospital, London, UK, in accordance with the Declaration of Helsinki and its amendments, the International Council for Harmonization Good Clinical Practice guidelines. It was approved by the UK Medicines and Healthcare products Regulatory Agency. The study protocol and informed consent form were reviewed and approved by the EDI Research and Ethics Committee and the Quorn Research Review Committee (approval nos. 03/E010 and QRRC03103/SRX-008). Written informed consent was obtained from all participants prior to enrollment. 

Subjects were required to be in good general health. They were ineligible if they had uncontrolled chronic disease that would contraindicate study participation; raised intracranial pressure, epilepsy, or a history of seizure disorder (except juvenile febrile seizures >10 years ago); a recognized risk of seizure; aminotransferases >2 times the upper limit of normal, or creatinine >170 µmol/L, or any other laboratory abnormality that contraindicated study participation; chronic analgesic or tranquilizer use or known substance abuse in the last 90 days; use of alcohol <12 hours prior to surgery; and use of the following within 14 days: monoamine oxidase inhibitors, tricyclic antidepressants (TCA), doxepin, neuroleptics, anorectics, bupropion, carbamazepine, quinidine, selective serotonin reuptake inhibitors (SSRIs), serotonin-norepinephrine reuptake inhibitors (SNRIs), tramadol, other opioids, antihistamines, tranquilizers, sedatives, or hypnotics.

## Case presentation

Case 1

The patient underwent an elective surgical removal of two third molars. Tooth 18 (FDI notation), the maxillary right third molar, was fully erupted, while tooth 48, the mandibular right third molar, exhibited a partial bony impaction. Both teeth were surgically extracted under local anesthesia. Four minutes after a single dose of IV tramadol 150 mg (Zydol®; Grunenthal Ltd.) on postsurgical day 1, this 30-year-old male subject with no known history of or risk factors for seizures experienced a generalized tonic-clonic seizure of severe intensity lasting five minutes. The seizures were considered by the investigator to be life-threatening and probably related to tramadol. Supportive care was given, including protection of the airway, suction, and oxygen by mask. The presurgical medical history and physical examination were unremarkable. Systolic and diastolic blood pressure (mm/Hg) and pulse (beats per minute) immediately before IV tramadol and at 0.25, 0.5, 1.0, and four hours post dose were 138/69/52, 156/87/102, 158/75//96, 132/75/95, and 130/68/61, respectively. Chemistry and hematology values were within normal limits at screening (preoperatively) and follow-up (following seizure). The subject did not report use of any medications associated with an increased risk of seizures in the 90 days prior to the study. Perioperative medications comprised chlorhexidine digluconate oral rinse and the local anesthetic, prilocaine hydrochloride 3%, in combination with the vasoconstrictor felypressin 0.54 µg/mL. He spontaneously recovered and was discontinued from the study due to the serious adverse event (SAE). The subject received acetaminophen and ibuprofen for postsurgical dental pain following the seizures. The recovery timeline is not available. No additional diagnostic work-up was done.

Case 2

The patient underwent an elective surgical removal of two third molars. Tooth 18, the maxillary right third molar, exhibited a complete bony impaction, while tooth 48, the mandibular right third molar, presented as a partial bony impaction. Both third molars were surgically extracted under local anesthesia. Three minutes after a single dose of IV tramadol 150 mg (Zydol®; Grunenthal Ltd.) on postsurgical day 1, this 23-year-old female subject with no known history of or risk factors for seizures experienced a generalized tonic-clonic seizure of severe intensity lasting three minutes. The seizures were considered by the Investigator to be life-threatening and probably related to tramadol. Supportive care was given, including protection of the airway, suction, and oxygen by mask. The presurgical medical history and physical examination were unremarkable. Systolic and diastolic blood pressure (mm/Hg) and pulse (beats per minute) immediately before IV tramadol and at 0.25, 0.5, 1.0, and four hours post dose were 131/84/61, 122/74/60, 122/71/63, 124/765/64, and 106/73/68. respectively. Chemistry and hematology values were within normal limits at screening (preoperatively) and follow-up (following seizure). The subject did not report use of any medications associated with an increased risk of seizures in the 90 days prior to the study. Perioperative medications comprised chlorhexidine digluconate oral rinse and the local anesthetic, prilocaine hydrochloride 3%, in combination with the vasoconstrictor felypressin of 0.54 µg/mL. The subject recovered spontaneously and was discontinued from the study due to the SAE. The subject received ibuprofen for postsurgical dental pain following the seizures. The recovery timeline is not available. No additional diagnostic work-up was done.

## Discussion

Tramadol was first approved for oral and IV administration in Europe in 1977 and 1991, respectively; oral, not IV, tramadol has been available in the US since 1995. Its widespread use is in part due to its dual mechanisms of action as a weak µ-opioid agonist and a monoaminergic reuptake inhibitor and because it was not subject to controlled substance regulations for several decades. Shortly after its introduction in the US, spontaneous adverse event reports submitted to the Food and Drug Administration (FDA) documented a marked incidence of seizures in individuals without a prior epilepsy diagnosis, often occurring within the recommended dose range and often following the first administration.

In 1996, the U.S. manufacturer of tramadol issued a “Dear Health Care Professional” letter warning of tramadol’s seizure risk, emphasizing that seizures occurred even at recommended doses, particularly when taken with SSRIs or TCAs [4,5]. The US prescribing information was also revised with stronger warnings about the potential for seizures. Despite this, reports of seizures continued to accumulate, prompting the FDA to further strengthen label warnings. In 2010, the FDA updated the Warnings section of tramadol’s prescribing information to emphasize the risk of overdose, addiction, and suicide in vulnerable patients, explicitly cautioning that tramadol-related deaths had occurred in patients prone to misuse or those taking tranquilizers or antidepressants concurrently. In 2014, reflecting the accumulating evidence of tramadol’s risks and to minimize overuse, abuse, and diversion into non-medical distribution channels, the U.S. Drug Enforcement Administration (DEA) classified tramadol as a Schedule IV controlled substance [6]. In the UK, it was similarly reclassified in 2014 as a controlled drug following concerns about increasing misuse and tramadol-related deaths.

Outside the US and Canada, IV Tramadol is widely used as monotherapy or as part of a multimodal analgesic regimen for the management of postsurgical pain. Since its initial approval, IV tramadol has been available in more than 135 countries. It is administered as a slow IV injection over two to three minutes or as an infusion. Both subjects had received a single IV tramadol dose of 150 mg over two minutes. Although IV tramadol 150 mg has been evaluated in prior studies involving healthy volunteers [7] and subjects with postsurgical pain [8-10], European prescribing guidance (Summary of Product Characteristics) recommends an initial 100 mg bolus, followed by 50 mg increments every 10 to 20 minutes as needed within the first hour, up to 250 mg, with maintenance dosing thereafter consisting of 50-100 mg every four to six hours, not to exceed 400 mg (previously 600 mg) daily [2,3].

The cause of tramadol-induced seizures is not well understood. Nevertheless, available nonclinical data suggest that some seizures following tramadol may be related to opioid-dependent GABA pathway and opioid-dependent histamine H1 receptor activation-linked mechanism [11-13]. The reported incidence of tramadol-associated seizures varies widely, based on the reporting country, commonly prescribed dose, concomitant use of proconvulsant drugs, and the availability and use of other analgesics, and local controlled substance regulations. A recent systematic review and meta-analysis of tramadol-associated seizures included 51 publications with a total sample size of 101,770 patients and reported the following seizure event rate in subgroups of tramadol poisoning, clinically indicated (therapeutic) use and non-medical use was 38% (95% CI: 27-49%), 3% (95% CI: 2-3%), and 37% (95% CI: 12-62%), respectively. The tramadol dose was significantly higher in the patients with seizures (mean differences: 0.82, CI 95%: 0.17-1.46). The odds for seizure occurrence were significantly associated with male sex [14].

The occurrence of seizures in two out of 22 subjects (9% of participants receiving IV tramadol at 150 mg) within four minutes of drug administration, despite rigorous screening for seizure risk factors, raises serious safety concerns. The seizures, deemed life-threatening by the investigator, led to Research Review Committee consultation, manufacturer notification, and termination of the IV tramadol 150 mg arm. For all analgesic efficacy outcomes, including pain intensity, pain relief, peak pain intensity, peak pain relief, time to rescue medication, and subject global evaluation, tramadol 150 mg was neither statistically nor numerically superior to tramadol 100 mg. In addition to seizures, the intensity of all adverse events with tramadol 150 mg was moderate or severe, while most adverse events with tramadol 100 mg were mild or moderate. Importantly, ketoprofen consistently outperformed tramadol; the efficacy of IV tramadol 100 mg and 150 mg was approximately half of that provided by IV ketoprofen 50 mg and 100 mg, with ketoprofen having mild or moderate adverse events comparable to placebo. 

## Conclusions

These findings suggest that for postsurgical pain (i) when compared to NSAIDs, such as ketoprofen, tramadol provides significantly lower analgesic efficacy; (ii) unless contraindicated, monotherapy with NSAIDs may be associated with a lower incidence and severity of adverse events; (iii) tramadol or pure µ-opioid receptor agonists may be more appropriate as part of multimodal analgesic regimens; and (iv) even a single bolus dose of IV tramadol 100 mg may provide an insufficient safety margin and pose an unacceptable risk of seizures in the real-world clinical setting. Accordingly, we recommend that IV tramadol for postsurgical pain be administered to adults either as a low-dose, repeated bolus (e.g., 25 to 50 mg every five to 10 minutes) or via a controlled infusion when doses exceed 50 mg, preferably in combination with a mechanistically differentiated analgesic.
